# Prognostic value of tissue-type plasminogen activator (tPA) and its complex with the type-1 inhibitor (PAI-1) in breast cancer

**DOI:** 10.1038/sj.bjc.6690353

**Published:** 1999-04

**Authors:** J H de Witte, C G J Sweep, J G M Klijn, N Grebenschikov, H A Peters, M P Look, ThH van Tienoven, J J T M Heuvel, J Bolt-De Vries, ThJ Benraad, J A Foekens

**Affiliations:** Department of Chemical Endocrinology, University Hospital Nijmegen, Geert Grooteplein 8, PO Box 9101, 6500 HB Nijmegen, The Netherlands; Division of Endocrine Oncology (Department of Medical Oncology), Rotterdam Cancer Institute (Dr Daniel den Hoed Kliniek)/Academic Hospital Rotterdam, Rotterdam, The Netherlands

**Keywords:** tissue-type plasminogen activator, tPA:PAI-1 complex, ELISA, cytosol, breast cancer, prognostic impact

## Abstract

The prognostic value of tissue-type plasminogen activator (tPA) measured in samples derived from 865 patients with primary breast cancer using a recently developed enzyme-linked immunosorbent assay (ELISA) was evaluated. Since the assay could easily be adapted to the assessment of the complex of tPA with its type-1 inhibitor (PAI-1), it was investigated whether the tPA:PAI-1 complex also provides prognostic information. To this end, cytosolic extracts and corresponding detergent extracts of 100 000 *g* pellets obtained after ultracentrifugation when preparing the cytosolic fractions for routine steroid hormone receptor determination were assayed. Statistically significant correlations were found between the cytosolic levels and those determined in the pellet extracts (Spearman correlation coefficient *r*_s_ = 0.75, *P* < 0.001 for tPA and *r* = 0.50, *P* < 0.001 for tPA:PAI-1 complex). In both Cox univariate and multivariate analysis elevated levels of (total) tPA determined in the pellet extracts, but not in cytosols, were associated with prolonged relapse-free (RFS) and overall survival (OS). In contrast, high levels of the tPA:PAI-1 complex measured in cytosols, but not in the pellet extracts, were associated with a poor RFS and OS. The prognostic information provided by the cytosolic tPA:PAI-1 complex was comparable to that provided by cytosolic (total) PAI-1. Furthermore, the estimated levels of free, uncomplexed tPA and PAI-1, in cytosols and in pellet extracts, were related to patient prognosis in a similar way as the (total) levels of tPA and PAI-1 respectively. Determination of specific forms of components of the plasminogen activation system, i.e. tPA:PAI-1 complex and free, uncomplexed tPA and/or PAI-1, may be considered a useful adjunct to the analyses of the separate components (tPA and/or PAI-1) and provide valuable additional prognostic information with respect to survival of breast cancer patients. © 1999 Cancer Research Campaign


*British Journal of Cancer* (1999) **80**(1/2), 286–294
© 1999 Cancer Research Campaign
Article no. bjoc.1998.0353

### Prognostic value of tissue-type plasminogen activator (tPA) and its complex with the type-1 inhibitor (PAI-1) in breast cancer

**JH de Witte[1], CGJ Sweep[1], JGM Klijn[2], N Grebenschikov[1], HA Peters[2], MP Look[2], ThH van Tienoven[1], JJTM Heuvel[1], J Bolt-De Vries[2], ThJ Benraad[1] and JA Foekens[2]**

[1]Department of Chemical Endocrinology, University Hospital Nijmegen, Geert Grooteplein 8, PO Box 9101, 6500 HB Nijmegen, The Netherlands; [2]Division of Endocrine Oncology (Department of Medical Oncology), Rotterdam Cancer Institute (Dr Daniel den Hoed Kliniek)/Academic Hospital Rotterdam, Rotterdam, The Netherlands

**Summary** The prognostic value of tissue-type plasminogen activator (tPA) measured in samples derived from 865 patients with primary breast cancer using a recently developed enzyme-linked immunosorbent assay (ELISA) was evaluated. Since the assay could easily be adapted to the assessment of the complex of tPA with its type-1 inhibitor (PAI-1), it was investigated whether the tPA:PAI-1 complex also provides prognostic information. To this end, cytosolic extracts and corresponding detergent extracts of 100 000 *g* pellets obtained after ultracentrifugation when preparing the cytosolic fractions for routine steroid hormone receptor determination were assayed. Statistically significant correlations were found between the cytosolic levels and those determined in the pellet extracts (Spearman correlation coefficient $r_s = 0.75$, $P < 0.001$ for tPA and $r = 0.50$, $P < 0.001$ for tPA:PAI-1 complex). In both Cox univariate and multivariate analysis elevated levels of (total) tPA determined in the pellet extracts, but not in cytosols, were associated with prolonged relapse-free (RFS) and overall survival (OS). In contrast, high levels of the tPA:PAI-1 complex measured in cytosols, but not in the pellet extracts, were associated with a poor RFS and OS. The prognostic information provided by the cytosolic tPA:PAI-1 complex was comparable to that provided by cytosolic (total) PAI-1. Furthermore, the estimated levels of free, uncomplexed tPA and PAI-1, in cytosols and in pellet extracts, were related to patient prognosis in a similar way as the (total) levels of tPA and PAI-1 respectively. Determination of specific forms of components of the plasminogen activation system, i.e. tPA:PAI-1 complex and free, uncomplexed tPA and/or PAI-1, may be considered a useful adjunct to the analyses of the separate components (tPA and/or PAI-1) and provide valuable additional prognostic information with respect to survival of breast cancer patients.

**Keywords:** tissue-type plasminogen activator; tPA:PAI-1 complex; ELISA; cytosol; breast cancer; prognostic impact

The plasminogen activation system represents a complex enzymatic cascade which plays a key role in fibrinolysis, tissue remodelling and invasive processes, including cancer (Danø et al, 1985; Rijken, 1995). Central to this system is the conversion of the inactive pro-enzyme plasminogen to plasmin, which is able to dissolve fibrin clots, degrade extracellular matrix proteins and activate latent pro-metalloproteases (Danø et al, 1985; Conese and Blasi, 1995; Rijken, 1995). Two enzymes, i.e. urokinase-type plasminogen activator (uPA) and tissue-type plasminogen activator (tPA), are both efficient activators of plasminogen. Unlike uPA, tPA has a strong affinity for fibrin, the presence of which strongly enhances tPA activity (Mignatti and Rifkin, 1993). Thus, both plasminogen activators have different physiological roles, uPA mediating tissue remodelling processes in a variety of normal and pathological conditions, whereas tPA is primarily involved in thrombolysis (Danø et al, 1985; Mignatti and Rifkin, 1993; Rijken, 1995). The activity of uPA and tPA is controlled in part by two main specific inhibitors, plasminogen activator inhibitor type-1 (PAI-1) and type-2 (PAI-2), which form inactive complexes of 1:1 stoichiometry with the plasminogen activators (Conese and Blasi, 1995; Rijken, 1995). Both uPA and PAI-1 are established prognostic factors in several types of cancer, a high tissue level of both components generally being associated with tumour progression and with poor disease-free and/or overall survival (reviewed by Duffy, 1996; Schmitt et al, 1997). Furthermore, several investigators have reported on the potential diagnostic and prognostic relevance of tPA analysed either in plasma or in tumour tissues from patients, including those afflicted with breast or colon cancer (Layer et al, 1987; De Bruin et al, 1988; Duffy et al, 1988; Needham et al, 1988; Grøndahl-Hansen et al, 1990; Jänicke et al, 1990, 1991; Rella, et al, 1993; Yamashita et al, 1993, 1995; Duggan et al, 1997). In addition, some studies even have examined the presence of complexes between tPA and PAI-1 in plasma from healthy individuals and patients with various diseases (Niwano et al, 1992; Hansson et al, 1994; Maser et al, 1997). In these different studies referred to above, ELISA methods have mostly been employed. Recently, a new ELISA suitable for the assessment of tPA in breast tumour tissue was developed (Grebenschikov et al, 1997). This ELISA, based on a combination of polyclonal antibodies, is easily adaptable to the assessment of its complex with PAI-1. In the present study, we applied the ELISA to the determination of tPA and tPA:PAI-1 complex in breast tumour cytosols and detergent extracts of the corresponding high-speed pellets. The prognostic relevance of the levels of tPA and of the tPA:PAI-1

*Received 13 July 1998*
*Revised 2 November 1998*
*Accepted 11 November 1998*

*Correspondence to*: JH de Witte

**Table 1** Relationships of tPA and tPA:PAI-1 complex levels in 865 cytosols and pellet extracts with patient and tumour characteristics

| Characteristics | Number of patients[a] | Percentage of tumours above the median value[b] | | | |
|---|---|---|---|---|---|
| | | $tPA_c$[c] | $tPA_p$[c] | tPA:PAI-$1_c$[c] complex | tPA:PAI-$1_p$[c] complex |
| All patients | 865 | 50 | 50 | 50 | 50 |
| Age at surgery (years) | | | | | |
| ≤ 40 | 117 | 33 | 31 | 48 | 41 |
| 41–55 | 297 | 54 | 56 | 46 | 46 |
| 56–70 | 291 | 48 | 48 | 53 | 55 |
| > 70 | 160 | 57 | 56 | 53 | 54 |
| *P*-value[d] | | 0.004 | 0.005 | 0.008 | 0.009 |
| Menopausal status | | | | | |
| Pre-menopausal | 353 | 51 | 49 | 47 | 44 |
| Post-menopausal | 512 | 49 | 51 | 52 | 54 |
| *P*-value[e] | | 0.80 | 0.50 | 0.02 | 0.004 |
| Tumour size | | | | | |
| $T_1$ (≤ 2 cm) | 395 | 53 | 58 | 51 | 53 |
| $T_2$ (> 2–5 cm) | 389 | 48 | 44 | 50 | 48 |
| $T_{3/4}$ (> 5 cm) | 81 | 42 | 38 | 47 | 45 |
| *P*-value[f] | | < 0.001 | < 0.001 | 0.79 | 0.01 |
| Nodal status | | | | | |
| $N_0$ | 434 | 50 | 55 | 48 | 50 |
| $N_{1–3}$ | 209 | 50 | 48 | 55 | 54 |
| $N_{>3}$ | 213 | 48 | 43 | 49 | 45 |
| *P*-value[f] | | 0.23 | < 0.001 | 0.45 | 0.07 |
| Grade | | | | | |
| Well/moderate | 151 | 56 | 62 | 49 | 51 |
| Poor | 496 | 44 | 45 | 51 | 52 |
| *P*-value[e] | | < 0.001 | < 0.001 | 0.81 | 0.67 |
| ER-positive[g] | | | | | |
| No | 201 | 18 | 18 | 38 | 35 |
| Yes | 663 | 59 | 60 | 53 | 54 |
| *P*-value[d] | | < 0.001 | < 0.001 | < 0.001 | < 0.001 |
| PgR-positive[g] | | | | | |
| No | 240 | 24 | 25 | 42 | 44 |
| Yes | 611 | 60 | 60 | 53 | 52 |
| *P*-value[d] | | < 0.001 | < 0.001 | < 0.001 | 0.002 |

[a]Due to missing values the numbers do not always add up to 865; values for tPA:PAI-$1_c$ and tPA:PAI-$1_p$ correspond to 782 and 822 patients, respectively. [b]Median antigen levels for $tPA_c$, $tPA_p$, tPA:PAI-$1_c$ and tPA:PAI-$1_p$ as indicated in the legend to Table II. [c]$tPA_c$, $tPA_p$, tPA:PAI-$1_c$ and tPA:PAI-$1_p$ denotes tPA and tPA:PAI-1 complex levels determined with the different ELISAs in cytosols (c) and pellet extracts (p), respectively. [d]*P*-value for Spearman rank correlation. [e]*P*-value for Wilcoxon rank-sum test (for grade: well and moderate combined). [f]*P*-value for Kruskal–Wallis test, including a Wilcoxon-type test for trend. [g]Cut-off point used for ER and PgR: 10 fmol mg$^{-1}$ protein.

complex in both types of samples was subsequently evaluated. Furthermore, we analysed the prognostic value of the calculated PAI-1 to tPA ratio and of the free, uncomplexed forms of both PAI-1 and tPA.

### MATERIALS AND METHODS

### Patients and tumour characteristics

This study was performed on a group of 865 patients with operable primary breast cancer in whom tPA antigen levels were measured in both cytosols and pellet extracts. The patients, whose samples were stored in the tumour bank of the Rotterdam Cancer Institute (Dr Daniel den Hoed Kliniek), included those for which tumour levels of uPA and PAI-1 were determined previously (De Witte et al, 1998). Patients with primary diagnosis of breast cancer between 1979 and 1989, without evidence of distant metastasis at the time of diagnosis, were included in the study. Patients with previous diagnosis of carcinoma, except for basal cell skin cancer or cervical cancer stage Ia, or with evidence of disease within 1 month after primary surgery were excluded. None of the patients received neo-adjuvant treatment before primary surgery. In case of mastectomy for residual disease after an initial lumpectomy, the mastectomy is considered (as part of) primary treatment. The median number of lymph nodes removed surgically was 11. Median age of the patients at the time of surgery was 56 years (range 25–89 years). Radiotherapy was given to 82% of the patients: on the breast/thoracic wall to 596 patients and/or on the axilla to 250 patients, and/or on one or more lymph node areas other than the axilla to 283 patients. Two of the 434 node-negative patients received adjuvant chemotherapy (CMF-cyclophosphamide, methotrexate, 5-fluorouracil), while none of these patients received adjuvant endocrine therapy. Of the 422 node-positive patients (for nine patients, nodal status missing), adjuvant chemotherapy was given to 138 patients, while 59 patients received hormonal therapy, either alone (45 patients) or in

© Cancer Research Campaign 1999 

combination with chemotherapy (14 patients). All patients were routinely examined every 3–6 months during the first 5 years of follow-up and once a year thereafter (median follow-up period of patients alive, 100 months; range, 12–167 months). During follow-up, 354 patients (41%) showed relapse and counted as failures in the analysis for relapse-free survival (RFS). Sixty-two patients (7%) died without evidence of disease and were censored at last follow-up in the analysis for RFS. Two-hundred and fourteen patients (25%) died after a previous relapse. A total of 276 (62+214) patients (32%) were counted as failures in the analysis for overall survival (OS). Further characteristics of patients and tumours are listed in Table 1.

### Tumour tissue extraction

Tumour tissues (stored in liquid nitrogen) were processed in the frozen state by pulverization with a microdismembrator and the resulting tissue powders homogenized in buffer (10 mM $K_2HPO_4$, containing 1.5 mM di-potassium chloride EDTA, 3 mM sodium azide, 10 mM monothioglycerol and 10% (v/v) glycerol, pH = 7.4), as recommended by the European Organization for Research and Treatment of Cancer for analysis of steroid hormone receptors (oestrogen (ER and PgR) (EORTC Breast Cancer Cooperative Group, 1980). The homogenates were centrifuged for 30 min at 100 000 ***g*** and 4°C to obtain the supernatant fractions (cytosols). The 100 000 ***g*** pellets were re-homogenized with an Ultraturrax tissue homogenizer in 20 mM Tris-HCl (pH = 8.5) containing 125 mM sodium chloride, after complete removal of the 50% (v/v) glycerol under which the pellets were interimly stored at –80°C. After adding Triton X-100 to a final concentration of 1%, the homogenates (total volume 1500 µl) were subsequently incubated for 16–20 h at 4°C under gentle shaking. The supernatant fractions obtained by centrifugation at 30 000 ***g*** at 4°C were designated as pellet extracts.

### Steroid hormone receptor assays

ER and PgR levels were determined by ligand binding assay or enzyme immunoassay in cytosols as described earlier (Foekens et al, 1989). The cut-off level used as to classify tumours as ER or PgR positive or negative was 10 fmol $mg^{-1}$ cytosolic protein.

### ELISAs

### *ELISA for tPA*

The antigen levels of tPA in cytosols and pellet extracts were assessed with a newly established ELISA (Grebenschikov et al, 1997). Briefly, microtitreplates were first coated overnight at 4°C with sheep anti-chicken IgY antibodies and then incubated with the specific chicken antibodies directed against tPA. Bound ligand was detected by incubation with rabbit anti-tPA antibodies and subsequent incubation with horseradish peroxidase (HRP)-labelled goat anti-rabbit antibodies (Sigma Chemical Co, St Louis, MO, USA) diluted 1:23 000. Recombinant single-chain tPA (Boehringer Ingelheim, Alkmaar, The Netherlands) was used as a standard in the ELISA. The tPA ELISA detects both the free form of tPA, as well as its complex with PAI-1 and PAI-2.

### *ELISA for tPA:PAI-1 complex*

The tPA:PAI-1 complex in cytosols and pellet extracts was determined in assay formats based on a combination of polyclonal antibodies employed in the ELISAs for the separate components, i.e. tPA and PAI-1 (Grebenschikov et al, 1997). Briefly, the chicken anti-tPA and rabbit anti-PAI-1 antibody (ELISA format A), and/or the chicken anti-PAI-1 and rabbit anti-tPA antibody (ELISA format B) were combined as catching and tagging antibody in one and the same ELISA frame for assessment of tPA:PAI-1 complex. Detection of complexes was performed with HRP-labelled goat anti-rabbit antibodies as described above. The tPA:PAI-1 complex standard was a gift from Prof. Andreasen (University of Århus, Århus, Denmark), prepared from the separate components as described previously (Egelund et al, 1997). The tPA:PAI-1 complex in cytosols and pellet extracts was determined essentially in both ELISA formats, except for those cases in which high levels of PAI-1 or tPA were to be expected; in these cases only format A ([PAI-1]≥10*[tPA]) or format B ([tPA]≥10*[PAI-1]) was chosen, thereby anticipating an underestimation of complex assessment because of possible competition of (excess) uncomplexed PAI-1 or tPA with tPA:PAI-1 complex for binding to the catching antibodies. At least two different dilutions of each patient sample were analysed in ELISA format A and/or format B, and the final concentration was calculated by extrapolation to infinite dilution using linear regression. When applying both ELISA formats, the highest antigen value obtained was considered as representing the actual tPA:PAI-1 complex concentration in the patient's sample. Strong statistically significant correlations were found between the antigen values obtained in ELISA format A and format B ($r_s = 0.97$, $P < 0.001$, for both the cytosols and the pellet extracts). The calculated percentage of tPA being in complex with PAI-1, the antigen levels expressed in molars, varied to a large extent, both in cytosols (median: 44%; inter-quartile range: 18–90%) and in pellet extracts (34%; 12–83%).

ELISA reagents were all diluted in phosphate-buffered saline (PBS) with 1% bovine serum albumin (BSA) and 0.1% (v/v) Tween-20. Incubations with standards, samples and controls, diluted in PBS-BSA-Tween, were performed overnight at 4°C. All determinations were performed in duplicate. Triton X-100 did not influence the ELISA results up to a concentration of 1%. The reproducibility of assay performance was controlled by analysis of an aliquot of a pooled breast tumour cytosol sample (tPA ELISA) or a pooled plasma sample (tPA:PAI-1 ELISA) in each assay-run and the between-assay variation was found to be below 10% for both ELISAs. The within-assay variation of samples measured in duplicate was always below 5%.

### Protein determinations

The Bradford method for protein analysis (Bradford, 1976) was employed using the Bio-Rad reagent with human serum albumin (KabiVitrum, Stockholm, Sweden) as a standard in order to express antigen levels per mg of total protein. Triton X-100 up to a concentration of 1% did not interfere with the protein determination in pellet extracts.

### Statistical analysis

The strength of the associations of tPA and tPA:PAI-1 complex levels determined in cytosols and pellet extracts with each other, age and steroid hormone receptor levels was tested with Spearman rank correlation ($r_s$). The associations of tPA and tPA:PAI-1 complex with other clinical variables were tested with the

© Cancer Research Campaign 1999

non-parametric Wilcoxon rank-sum test or the Kruskal–Wallis test, including a Wilcoxon-type test for trends across ordered groups where appropriate. RFS and OS probabilities were calculated by the actuarial method of Kaplan and Meier (Kaplan and Meier, 1958). The log-rank test for trend was used to test ordered variables. Both uni- and multivariate analyses were performed using the Cox proportional hazard model, and the associated likelihood ratio test was used to test for differences. All computations were done with the STATA statistical package, release 5.0 (STATA Corp., College Station, TX, USA). Two-sided $P$-values below 0.05 were considered to be statistically significant.

### RESULTS

### tPA and tPA:PAI-1 complex in cytosols and pellet extracts

The antigen levels of tPA determined in the cytosols varied from 0.02 to 216.75 ng mg$^{-1}$ protein (mean 6.38; median 2.40) and those in the pellet extracts varied from 0.06 to 2263.00 ng mg$^{-1}$ protein (mean 38.19; median 13.01). The cytosolic levels of tPA:PAI-1 complex ranged from 0.18 to 30.04 ng mg$^{-1}$ protein (mean 2.34; median 1.75) and in the pellet extracts from 0.20 to 374.00 (mean 9.91; median 6.57). As compared to the cytosolic levels of the respective antigens, the levels determined in the pellet extracts were approximately sixfold (tPA) and fourfold (tPA:PAI-1 complex) higher. However, the correlations between the levels in cytosols and in the corresponding pellet extracts were statistically significant ($r_s = 0.75$, $P < 0.001$ for tPA and $r_s = 0.50$, $P < 0.001$ for tPA:PAI-1 complex). Significant correlations were also found between the levels of tPA and tPA:PAI-1 complex in cytosols ($r_s = 0.30$, $P < 0.001$) and pellet extracts ($r_s = 0.25$, $P < 0.001$).

### Relation of tPA and tPA:PAI-1 complex to patient and tumour characteristics

The levels of tPA and tPA:PAI-1 complex determined in cytosols and pellet extracts were related to patient and tumour characteristics (Table 1). The levels of tPA and tPA:PAI-1 complex measured in cytosols or pellet extracts were positively related with patient's age. The cytosolic level of tPA, as well as its level in pellet extracts, was not related with menopausal status, while those of tPA:PAI-1 complex were significantly higher in tumours from post-menopausal patients. In general, tPA or tPA:PAI-1 complex levels were negatively related with the size of the primary tumour. In contrast, the levels of tPA and tPA:PAI-1 complex, measured

**Table 2** Cox univariate and multivariate analyses of relapse-free and overall survival in 865 breast cancer patients

| Factor | Univariate analyses RFS | | Multivariate analyses RFS | | Univariate analyses OS | | Multivariate analyses OS | |
|---|---|---|---|---|---|---|---|---|
| | *P*-value | RHR (95% CI)[a] | *P*-value | RHR (95% CI)[b] | *P*-value | RHR (95% CI)[a] | *P*-value | RHR (95% CI)[b] |
| Age and menopausal status | < 0.001[c] | | < 0.001[c] | | < 0.001[c] | | < 0.001[c] | |
| Age/pre-menopausal[d] | | 0.59 (0.46–0.77) | | 0.63 (0.49–0.81) | | 0.74 (0.53–1.05) | | 0.81 (0.57–1.14) |
| Age/post-menopausal[d] | | 1.08 (0.92–1.26) | | 1.00 (0.85–1.17) | | 1.49 (1.27–1.76) | | 1.36 (1.15–1.61) |
| Post- vs pre-menopausal[e] | | 1.68 (1.09–2.59) | | 1.38 (0.88–2.16) | | 1.51 (0.87–2.61) | | 1.19 (0.67–2.10) |
| Tumour size | < 0.001 | | 0.08 | | < 0.001 | | < 0.001 | |
| 2–5 cm vs ≤ 2 cm | | 1.56 (1.24–1.95) | | 1.22 (0.96–1.56) | | 1.75 (1.34–2.29) | | 1.18 (0.88–1.58) |
| > 5 cm vs ≤ 2 cm | | 2.43 (1.72–3.42) | | 1.54 (1.04–2.28) | | 3.82 (2.69–5.40) | | 2.16 (1.46–3.21) |
| Nodal status | < 0.001 | | < 0.001 | | < 0.001 | | 0.004 | |
| $N_{1-3}$ vs $N_0$ | | 1.22 (0.93–1.61) | | 1.51 (1.09–2.09) | | 1.83 (1.34–2.51) | | 2.34 (1.64–3.34) |
| $N_{>3}$ vs $N_0$ | | 2.68 (2.11–3.41) | | 2.88 (2.12–3.91) | | 3.78 (2.86–5.00) | | 3.80 (2.71–5.35) |
| ER[f] | < 0.001 | 0.64 (0.51–0.81) | 0.05 | 0.75 (0.56–1.00) | < 0.001 | 0.57 (0.45–0.74) | 0.007 | 0.65 (0.48–0.88) |
| PgR[f] | 0.02 | 0.76 (0.61–0.95) | 0.83 | 0.97 (0.74–1.28) | < 0.001 | 0.59 (0.46–0.75) | 0.03 | 0.72 (0.53–0.96) |
| Adjuvant therapy[g] | 0.05 | 0.76 (0.58–1.00) | 0.007 | 0.65 (0.48–0.89) | < 0.001 | 0.53 (0.40–0.72) | 0.008 | 0.63 (0.44–0.89) |
| tPA *cytosol* | 0.06 | | 0.90[h] | | 0.07 | | 0.94[h] | |
| $Q_2$ vs $Q_1$ | | 0.81 (0.61–1.07) | | 0.99 (0.73–1.34) | | 0.81 (0.58–1.11) | | 1.11 (0.79–1.57) |
| $Q_3$ vs $Q_1$ | | 0.71 (0.53–0.96) | | 0.89 (0.64–1.24) | | 0.78 (0.56–1.08) | | 1.04 (0.72–1.50) |
| $Q_4$ vs $Q_1$ | | 0.78 (0.58–1.04) | | 0.98 (0.71–1.37) | | 0.73 (0.52–1.02) | | 1.04 (0.71–1.53) |
| tPA *pellet* | < 0.001 | | 0.06 | | < 0.001 | | 0.03 | |
| $Q_2$ vs $Q_1$ | | 0.76 (0.57–1.00) | | 0.86 (0.65–1.15) | | 0.76 (0.56–1.02) | | 0.95 (0.69–1.30) |
| $Q_3$ vs $Q_1$ | | 0.60 (0.45–0.81) | | 0.75 (0.55–1.04) | | 0.55 (0.39–0.76) | | 0.73 (0.51–1.05) |
| $Q_4$ vs $Q_1$ | | 0.55 (0.41–0.73) | | 0.64 (0.46–0.89) | | 0.48 (0.34–0.67) | | 0.60 (0.41–0.88) |
| tPA:PAI-1 complex *cytosol* | 0.01 | | 0.03 | | < 0.001 | | < 0.001 | |
| $Q_2$ vs $Q_1$ | | 1.27 (0.92–1.75) | | 1.26 (0.91–1.74) | | 1.81 (1.22–2.67) | | 1.91 (1.28–2.86) |
| $Q_3$ vs $Q_1$ | | 1.42 (1.03–1.95) | | 1.51 (1.09–2.09) | | 1.67 (1.12–2.47) | | 1.92 (1.28–2.88) |
| $Q_4$ vs $Q_1$ | | 1.47 (1.07–2.00) | | 1.59 (1.15–2.18) | | 2.25 (1.54–3.27) | | 2.62 (1.77–3.87) |
| tPA:PAI-1 complex *pellet* | 0.81 | | 0.35 | | 0.10 | | 0.03 | |
| $Q_2$ vs $Q_1$ | | 1.11 (0.82–1.50) | | 1.09 (0.80–1.48) | | 1.10 (0.78–1.56) | | 1.14 (0.80–1.64) |
| $Q_3$ vs $Q_1$ | | 1.09 (0.80–1.47) | | 1.15 (0.85–1.58) | | 1.20 (0.85–1.69) | | 1.35 (0.95–1.93) |
| $Q_4$ vs $Q_1$ | | 1.05 (0.77–1.42) | | 1.33 (0.97–1.83) | | 1.24 (0.89–1.75) | | 1.71 (1.19–2.45) |

[a]Relative hazard rate (RHR) with 95% confidence interval (CI) of univariate analyses. [b]Relative hazard rate (RHR) with 95% confidence interval (CI) of multivariate analyses (final model, 841 patients; patients with missing values for ER and PgR ($n$ = 15), and missing information on nodal status ($n$ = 9) not included) corrected for the basic model, including age/menopausal status, tumour size, nodal status, ER/PgR status, and adjuvant therapy; factors were added separately to the basic model. [c]Age and menopausal status combined. [d]Age in decades tested separately for pre- and post-menopausal patients. [e]Post-menopausal as compared with pre-menopausal. [f]Cut-off points used for ER and PgR, 10 fmol mg$^{-1}$ protein. [g]Adjuvant therapy (yes vs no) only for node-positive patients. [h]$P$-values for the associated likelihood ratio test for the quarters ($Q_1$–$Q_4$) indicated: $Q_1$, first quarter; $Q_2$, second quarter; $Q_3$, third quarter; $Q_4$, fourth quarter. The quartiles of the tPA antigen levels were successively 1.21, 2.40, 4.72 ng mg$^{-1}$ (for cytosols) and 5.48, 13.01, 33.18 ng mg$^{-1}$ (for pellet extracts); the quartiles of the tPA:PAI-1 complex levels were successively 0.97, 1.75, 3.08 ng mg$^{-1}$ (for cytosols) and 3.69, 6.57, 10.16 ng mg$^{-1}$ (for pellet extracts).

*© Cancer Research Campaign 1999*

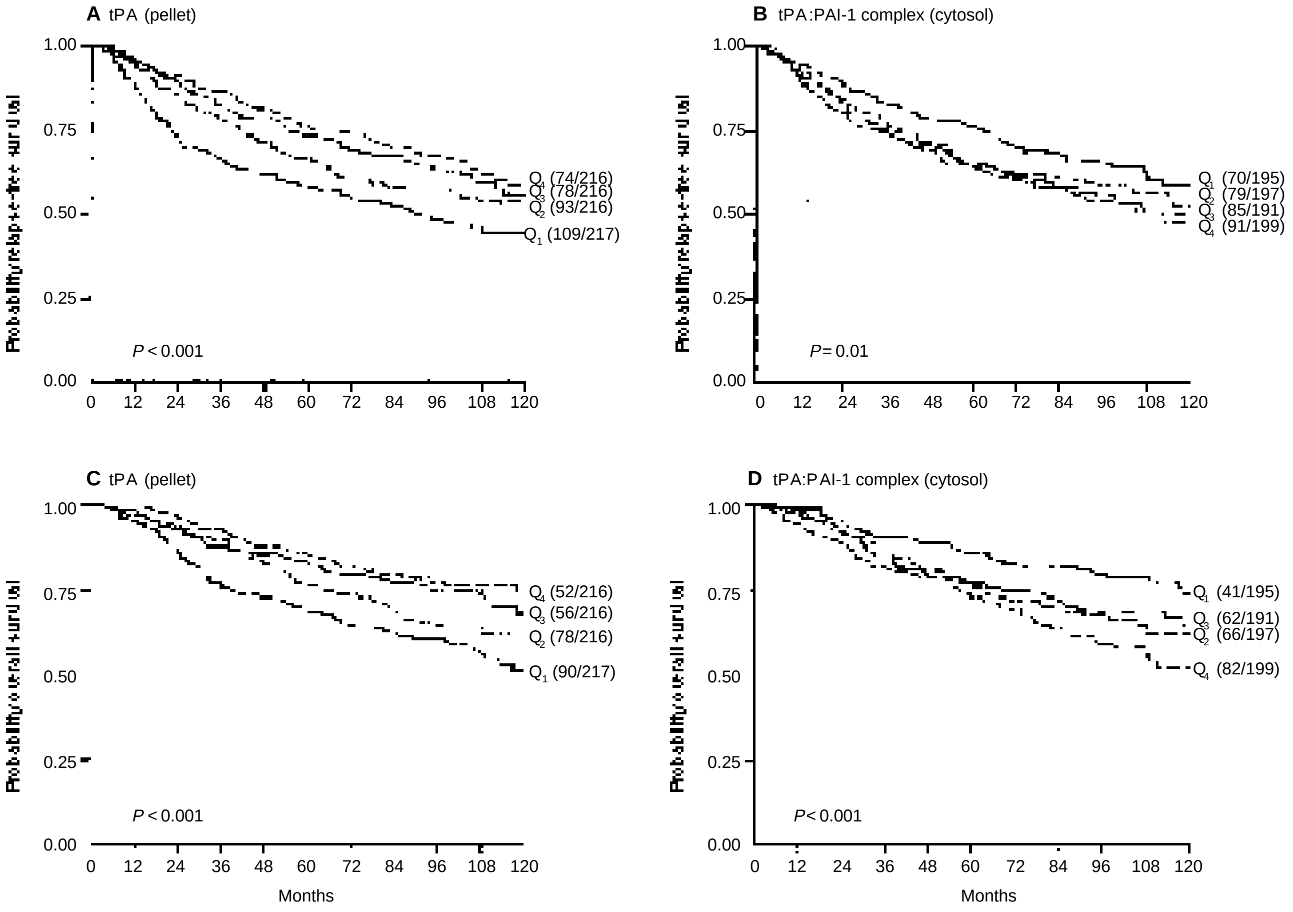

**Figure 1** Actuarial relapse-free and overall survival curves for breast cancer patients stratified by the levels of tPA in pellet extracts (**A** and **C**) and of tPA:PAI-1 complex in cytosols (**B** and **D**). Patients were divided into groups according to the quartiles ($Q_1$– $Q_4$) of the respective levels as indicated in the legend to Table 2

**Table 3** Cox univariate and multivariate analyses of relapse-free and overall survival in 865 breast cancer patients

| Factor | Univariate analyses RFS | | Multivariate analyses RFS | | Univariate analyses OS | | Multivariate analyses OS | |
|---|---|---|---|---|---|---|---|---|
| | *P*-value | RHR (95% CI)[a] | *P*-value[c] | RHR (95% CI)[b] | *P*-value | RHR (95% CI[a] | *P*-value[c] | RHR (95% CI)[b] |
| PAI-1 *cytosol* | < 0.001 | | < 0.001 | | < 0.001 | | < 0.001 | |
| $Q_2$ vs $Q_1$ | | 1.15 (0.84–1.59) | | 1.31 (0.94–1.81) | | 1.23 (0.83–1.81) | | 1.42 (0.95–2.13) |
| $Q_3$ vs $Q_1$ | | 1.50 (1.10–2.04) | | 1.55 (1.13–2.13) | | 1.73 (1.20–2.49) | | 1.80 (1.23–2.63) |
| $Q_4$ vs $Q_1$ | | 2.05 (1.53–2.76) | | 2.12 (1.55–2.88) | | 2.61 (1.85–3.69) | | 2.69 (1.87–3.88) |
| PAI-1 *pellet* | < 0.001 | | 0.001 | | < 0.001 | | < 0.001 | |
| $Q_2$ vs $Q_1$ | | 1.02 (0.75–1.39) | | 1.08 (0.78–1.49) | | 0.95 (0.66–1.38) | | 1.10 (0.75–1.62) |
| $Q_3$ vs $Q_1$ | | 1.35 (1.00–1.83) | | 1.43 (1.05–1.95) | | 1.45 (1.03–2.05) | | 1.60 (1.12–2.29) |
| $Q_4$ vs $Q_1$ | | 1.57 (1.17–2.12) | | 1.75 (1.28–2.38) | | 1.91 (1.36–2.67) | | 2.19 (1.53–3.12) |
| PAI-1/tPA *cytosol* | < 0.001 | | 0.06 | | < 0.001 | | < 0.004 | |
| $Q_2$ vs $Q_1$ | | 0.90 (0.66–1.23) | | 1.04 (0.75–1.43) | | 0.77 (0.52–1.13) | | 0.84 (0.57–1.25) |
| $Q_3$ vs $Q_1$ | | 1.15 (0.85–1.56) | | 1.07 (0.78–1.47) | | 1.45 (1.03–2.03) | | 1.27 (0.88–1.81) |
| $Q_4$ vs $Q_1$ | | 1.63 (1.22–2.18) | | 1.47 (1.07–2.02) | | 1.85 (1.33–2.57) | | 1.64 (1.13–2.36) |
| PAI-1/tPA *pellet* | < 0.001 | | 0.004 | | < 0.001 | | < 0.001 | |
| $Q_2$ vs $Q_1$ | | 1.04 (0.75–1.43) | | 1.22 (0.88–1.69) | | 0.98 (0.66–1.44) | | 1.21 (0.81–1.81) |
| $Q_3$ vs $Q_1$ | | 1.45 (1.06–1.97) | | 1.33 (0.97–1.83) | | 1.89 (1.33–2.67) | | 1.68 (1.17–2.43) |
| $Q_4$ vs $Q_1$ | | 1.87 (1.40–2.52) | | 1.82 (1.32–2.53) | | 2.15 (1.52–3.03) | | 2.05 (1.40–3.02) |

[a]Relative hazard rate (RHR) with 95% confidence interval (CI) of univariate analyses. [b]Relative hazard rate (RHR) with 95% confidence interval (CI) of multivariate analyses corrected for the basic model, including age/menopausal status, tumour size, nodal status, ER/PgR status, and adjuvant therapy; factors were added separately to the basic model. [c]*P*-values for the associated likelihood ratio test for the quarters ($Q_1$–$Q_4$) indicated; $Q_1$, first quarter; $Q_2$, second quarter; $Q_3$, third quarter; $Q_4$, fourth quarter. The quartiles of the PAI-1 antigen levels were successively 1.06, 1.62, 2.63 ng $mg^{-1}$ (for cytosols) and 3.30, 5.28, 8.61 ng $mg^{-1}$ (for pellet extracts); the quartiles of the PAI-1/tPA ratios were successively 0.27, 0.66, 1.65 ng $mg^{-1}$ (for cytosols) and 0.15, 0.41, 1.32 ng mg $^{-1}$ (for pellet extracts).

 *© Cancer Research Campaign 1999*

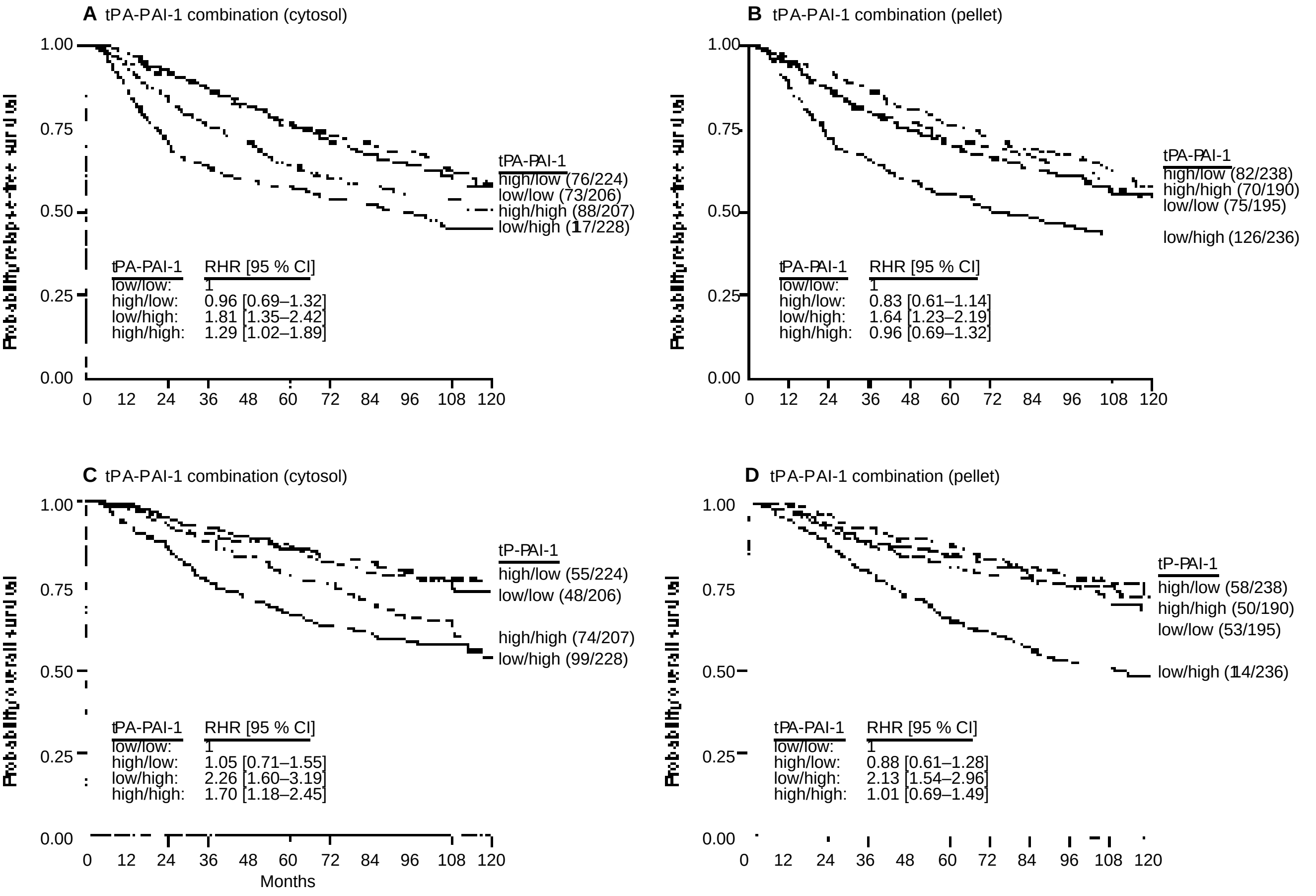

**Figure 2** Actuarial relapse-free and overall survival curves for breast cancer patients stratified by the combined levels of tPA and PAI-1 determined in cytosols (**A** and **C**) and in pellet extracts (**B** and **D**). Patients were divided into groups with levels below ('low') and above ('high') the median value of the respective tPA and PAI-1 levels as indicated in the legend to Table 1 and Table 3

**Table 4** Cox univariate and multivariate analyses of relapse-free and overall survival in 865 breast cancer patients

| Factor | Univariate analyses RFS | | Multivariate analyses RFS | | Univariate analyses OS | | Multivariate analyses OS | |
|---|---|---|---|---|---|---|---|---|
| | *P*-value | RHR (95% CI)[a] | *P*-value[c] | RHR (95% CI)[b] | *P*-value | RHR (95% CI)[a] | *P*-value[c] | RHR (95% CI)[b] |
| $tPA_{free}$ *cytosol* | 0.02 | 0.78 (0.64–0.97) | 0.33 | 0.89 (0.71–1.12) | 0.003 | 0.70 (0.55–0.88) | 0.08 | 0.79 (0.61–1.03) |
| $tPA_{free}$ *pellet* | < 0.001 | 0.66 (0.54–0.82) | 0.02 | 0.76 (0.60–0.96) | < 0.001 | 0.58 (0.46–0.74) | 0.002 | 0.66 (0.51–0.87) |
| $PAI\text{-}1_{free}$ *cytosol* | < 0.001 | 1.52 (1.22–1.89) | 0.002 | 1.43 (1.13–1.79) | < 0.001 | 2.17 (1.68–2.81) | < 0.001 | 2.01 (1.53–2.63) |
| $PAI\text{-}1_{free}$ *pellet* | < 0.001 | 1.45 (1.18–1.80) | < 0.001 | 1.45 (1.16–1.82) | < 0.001 | 1.78 (1.40–2.28) | < 0.001 | 1.70 (1.32–2.20) |

[a]Relative hazard rate (RHR) with 95% confidence interval (CI) of univariate analyses. [b]Relative hazard rate (RHR) with 95% confidence interval (CI) of multivariate analyses corrected for the basic model, including age/menopausal status, tumour size, nodal status, ER/PgR status, and adjuvant therapy; factors were added separately to the basic model. [c]*P*-values for the associated likelihood ratio test for the median values of the levels of the free, uncomplexed forms of tPA ($tPA_{free}$) and PAI-1 ($PAI\text{-}1_{free}$). The median values in ng $mg^{-1}$ protein were: 1.74 for $tPA_{free}$ *cytosol*, 11.11 for $tPA_{free}$ *pellet*, 1.26 for $PAI\text{-}1_{free}$ *cytosol*, 3.68 for $PAI\text{-}1_{free}$ *pellet*.

either in cytosols or pellet extracts, were not related with nodal status, except for tPA in pellet extracts, its levels decreasing with advanced lymph node involvement. The levels of tPA determined in cytosols and pellet extracts were significantly lower in poorly differentiated tumours, whereas no relations were found between the levels of tPA:PAI-1 complex and tumour grade. Overall, higher levels of tPA and tPA:PAI-1 complex were measured in steroid hormone-positive tumours, the correlation coefficients ($r_s$) observed ranging between 0.10 and 0.42.

### Relation of tPA and tPA:PAI-1 complex to survival

In Cox univariate regression analysis, young pre-menopausal age, post-menopausal status, the size of the primary tumour, the number of positive lymph nodes, steroid hormone receptor-negativity and adjuvant therapy in node-positive patients were significantly associated with reduced RFS and OS (Table 2). When divided into four groups ($Q_1$ to $Q_4$) by their respective quartiles, decreasing levels of tPA in pellet extracts and increasing levels of

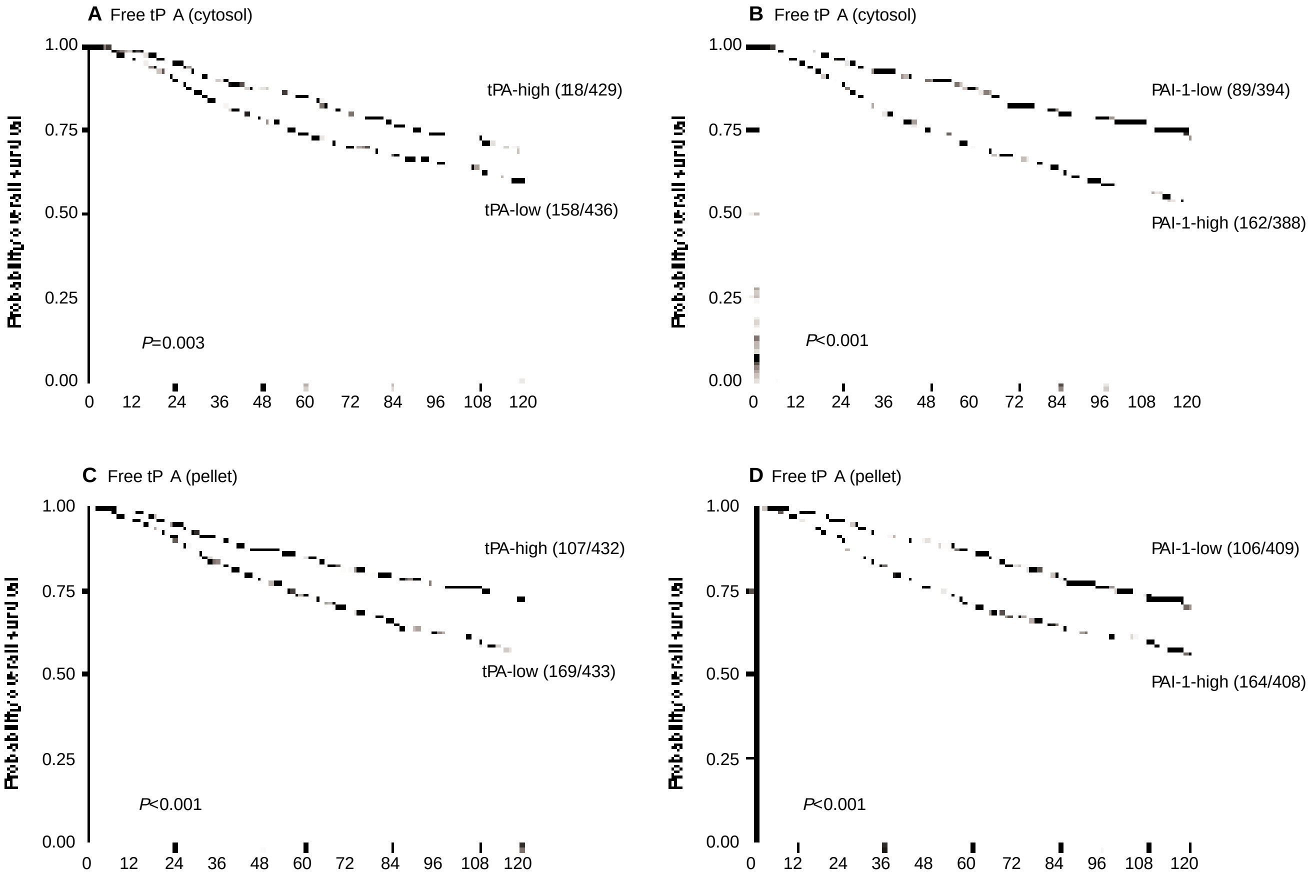

**Figure 3** Actuarial overall survival curves for breast cancer patients stratified by the estimated levels of free, uncomplexed tPA (**A** and **C**) and of free, uncomplexed PAI-1 (**B** and **D**) in cytosols and in pellet extracts. Patients were divided into groups according to the median values indicated in the legend to Table 4

tPA:PAI-1 complex in cytosols showed a clear trend towards a worse RFS and OS (Table 2, Figure 1). The levels of tPA in cytosols and those of tPA:PAI-1 complex in the pellet extracts were not significantly related with RFS and OS.

Cox multivariate regression analysis was performed to compare the prognostic significance of tPA and tPA:PAI-1 complex with that of the classical prognostic parameters (Table 2), these latter variables comprising a basic multivariate model. In the basic multivariate model, age, menopausal status, nodal status, ER status and adjuvant therapy were all significantly associated with both RFS and OS, nodal status being the strongest of the classical prognostic factors. Tumour size and PgR status were only statistically significant in predicting OS, not RFS (Table 2). Corrected for the basic multivariate model, the levels of tPA:PAI-1 complex in cytosols and pellet extracts were related with a poor RFS and OS, although this relationship for the pellet extracts with RFS was statistically not significant. The prognostic information provided by the tPA:PAI-1 complex appeared to be stronger in cytosols as compared to the pellet extracts. The levels of tPA did not significantly add to the basic multivariate model with respect to both RFS and OS, although, in the analysis for OS, the contribution of tPA in pellet extracts reached statistical significance ($P$ = 0.03) (Table 2).

Recently, the antigen levels of PAI-1 were determined in the same cytosols and pellet extracts, applying the same ELISA framework (De Witte et al, 1998). The (total) levels of PAI-1 and tPA in both types of extracts were very weakly negatively correlated with each other ($r_s$ = –0.073, $P$ = 0.033, for cytosols; $r_s$ = –0.12, $P$ = 0.001, for pellet extracts). Since both factors have opposite effects on patient prognosis, the ratio of PAI-1 to tPA (PAI-1/tPA) was calculated as an alternative variable to be related with survival. Both the antigen levels of PAI-1 and of the PAI-1: tPA ratio, either in cytosols or in pellet extracts, were associated with the rates of relapse and death, also when corrected for the basic multivariate model (Table 3). After including PAI-1 in the basic multivariate model, tPA did not further contribute to the prognostic information already provided by PAI-1. Figure 2 shows the actuarial RFS and OS curves of patients as a function of the combined effect of (total) tPA and (total) PAI-1 in cytosols and pellet extracts, both factors divided by their median value. Clearly, patients with low levels of tPA in their tumours, which, in addition, contain high levels of PAI-1 experienced a very poor prognosis. In turn, patients with both high tPA and low PAI-1 tumour levels encountered a favourable prognosis.

Finally, the levels of the free, uncomplexed forms of tPA and PAI-1 in cytosols and pellet extracts were estimated by subtraction

 *© Cancer Research Campaign 1999*

of the (molar) concentrations of the tPA:PAI-1 complexes from the (total) concentrations of tPA and PAI-1, respectively. In univariate analyses with the corresponding median values chosen to discriminate between low-risk and high-risk patients, high levels of uncomplexed tPA in cytosols and pellet extracts were significantly associated with prolonged RFS and OS (Table 4, Figure 3A, C). In contrast, high levels of uncomplexed PAI-1 in cytosols and pellet extracts appeared to be related with a significantly reduced RFS and OS (Table 4; Figure 3B, D). Moreover, in multivariate analyses corrected for the classical prognostic factors, uncomplexed PAI-1 in cytosols and in pellet extracts was found to be related with both poor RFS and OS, comparably strong as total PAI-1. The uncomplexed form of tPA measured in pellet extracts, but not in cytosolic extracts, added significantly to the basic multivariate model with respect to both RFS and OS (Table 4).

### DISCUSSION

In the present study, the prognostic impact of tPA antigen levels in a relatively large series of 865 breast cancer patients was evaluated employing a newly developed ELISA. The antigen levels were determined in cytosols routinely used for steroid hormone receptor analyses, as well as in extracts of 100 000 ***g*** pellets, which may be considered as incidental products of ultracentrifugation when preparing the cytosolic fractions. Its appearance in the pellet extracts may suggest the presence of a membrane-associated form of tPA. Indeed, receptors for tPA have been identified on the surfaces of vascular endothelial cells (Dudani and Ganz, 1996), which, in turn, are regarded as the primary source of tPA (Levin et al, 1997). In univariate analyses, high levels of tPA in pellet extracts were significantly associated with a lower probability of developing recurrences and of experiencing an early death. However, in multivariate analyses, after correction for the classical prognostic factors, elevated levels of tPA in pellet extracts appeared only as a weak predictor of improved OS ($P = 0.03$), but not RFS ($P = 0.06$). These findings resemble those obtained by Jänicke et al (1991) who determined tPA in detergent extracts of breast cancer tissue by ELISA, and showed that patients with a high tPA content tended to have a better prognosis than those with low, or no, detectable tPA content. However, the difference was not statistically significant. Furthermore, Duggan et al (1997), applying an ELISA to breast tumour extracts prepared with detergent-containing buffer, determined tPA to be an independent prognostic factor in breast cancer, with a calculated relative risk of relapse and of death corresponding to 0.56 and 0.47, respectively. Regarding the cytosolic extracts of breast tumour tissue, their tPA antigen levels did not have any prognostic impact on the rates of relapse or death. These findings are in apparent contrast to a previous report by Duffy et al (1988) who showed that patients with high cytosolic levels of tPA had a significantly longer disease-free interval and survival compared to patients with low tPA levels. The median follow-up period, however, was considerably shorter (26 months). In agreement with other investigations, the cytosolic levels of tPA were positively related to steroid hormone receptor (ER and/or PgR) status (Duffy et al, 1986; Needham et al, 1988; Schmitt et al, 1990; Jänicke et al, 1991; Rella et al, 1993). In this respect, the observed correlation of high tPA antigen levels with good prognosis may be related to the fact that tPA is an oestrogen-inducible enzyme (Butler et al, 1983; Mira-Y-Lopez and Ossowski, 1987), the presence of ER itself generally being associated with good prognosis in breast carcinomas (Table 2). Therefore, the presence of tPA may be directly representative of a functional, biologically active ER system, important for planning endocrine therapy, as has been suggested by Rella et al (1993). The positive clinical impact of tPA on survival is also expressed by the combined prognostic value of tPA and PAI-1 measured in cytosols but mainly in pellet extracts (Figure 2), exemplifying that tPA abolishes the unfavourable effect of PAI-1 on prognosis. An interesting finding of the present study concerns the prognostic impact of tPA:PAI-1 complex measured in the breast tumour cytosols on survival. In multivariate analyses, cytosolic tPA:PAI-1 complex provided prognostic information, which appeared to be almost as strong as cytosolic PAI-1 with respect to RFS and, especially, OS. This latter observation is in line with the supposition that the levels of tPA:PAI-1 complex in tumour tissue extracts may indirectly reflect the level of PAI-1 which has been originally active, since only the active form of PAI-1 is able to form complexes with tPA (Hekman and Loskutoff, 1985). Although PAI-1 was shown to be a strong independent prognostic parameter also in pellet extracts, tPA:PAI-1 complex did not have prognostic impact on RFS and was only a weak predictor of OS when measured in this type of extracts. Consequently, this latter finding may suggest that the prognostic relevance exerted by PAI-1 in the pellet extracts does not originate from its complex with tPA, but may be provided by the free, uncomplexed form of PAI-1. Presumably, this also accounts for the prognostic significance of (free, uncomplexed) tPA in the pellet extracts (see below). From an analytical–chemical point of view, the ELISA for tPA:PAI-1 complex can be considered as being highly specific for the component to be assessed. While the tPA ELISA detects both the free and complexed forms of tPA, the tPA:PAI-1 complex ELISA selectively quantifies only one molecular variant of tPA, i.e. its complex with PAI-1, without being sensitive towards the separate components, tPA and PAI-1 (Grebenschikov et al, 1997). Moreover, the results obtained with the tPA:PAI-1 complex ELISA may be regarded as being highly reliable, since the tPA:PAI-1 complex standard used for quantitation of the assay results is directly comparable to the analyte to be assessed. In contrast, the single-chain recombinant tPA standard employed in the tPA ELISA, clearly is not fully representative of the different molecular forms of tPA present in the clinical samples (see below), a complication inherently linked to assays attempting to quantify a heterogenous component (Benraad et al, 1996).

The PAI-1/tPA ratio, introduced as an alternative parameter, also provided strong prognostic information, high levels in cytosols as well as in pellet extracts predicting both reduced RFS and OS. Most likely, a high PAI-1:tPA ratio is representative of a large excess of (free, uncomplexed) PAI-1 predictive of poor prognosis, while a low PAI-1/tPA ratio reflects a relative excess of (free, uncomplexed) tPA with a favourable effect on prognosis. This consideration may underlie the observed associations of high levels of the free, uncomplexed forms of both PAI-1 and tPA in cytosols and pellet extracts with successively a poor and good prognosis. However, one has to realize that the levels of the free, uncomplexed forms are just estimations based on subtraction of tPA:PAI-1 complex levels from the total antigen levels of tPA and PAI-1, the more so as other complexed forms such as tPA:PAI-2 and uPA:PAI-1 might also be detected in the ELISA for (total) tPA and (total) PAI-1, respectively. Moreover, as compared to the free, uncomplexed forms, each of the various complexes will be detected with different efficiencies in the standard ELISAs, which, in addition, make use of standards or calibrators (recombinant

© Cancer Research Campaign 1999

single-chain tPA and latent human PAI-1) which may not be completely representative of the clinical samples to be analysed. Notwithstanding these limitations, the observed impact of the free, uncomplexed forms on survival are in accordance with our expectations of a favourable (tPA) and an unfavourable (PAI-1) prognostic marker. Accordingly, the development of immunoassays for the selective detection of specific forms of the various components of the plasminogen activation system, i.e. tPA:PAI-1 complex, free uncomplexed tPA and/or free, uncomplexed PAI-1, may be a useful adjunct to the analyses of the separate components (tPA and/or PAI-1) by ELISA, the latter assay results representing at best the total antigen content of the clinical sample.

### REFERENCES

Benraad ThJ, Geurts-Moespot J, Grøndahl-Hansen J, Schmitt M, Heuvel JJTM, De Witte JH, Foekens JA, Leake RE, Brünner N and Sweep CGJ (1996) Immunoassays (ELISA) of urokinase-type plasminogen activator (uPA): report of an EORTC/BIOMED-1 workshop. *Eur J Cancer* **32A**: 1371–1381

Bradford MM (1976) A rapid and sensitive method for the quantitation of microgram quantities of protein utilizing the principle of protein-dye binding. *Anal Biochem* **72**: 248–254

Butler WB, Kirkland WL, Gargala TL, Goran N, Kelsey WH and Berlinsky PJ (1983) Steroid stimulation of plasminogen activator production in a human breast cancer cell line (MCF-7). *Cancer Res* **43**: 1637–1641

Conese M and Blasi F (1995) Urokinase/urokinase receptor system: internalization/degradation of urokinase-serpin complexes: mechanisms and regulation. *Biol Chem Hoppe-Seyler* **376**: 143–155

Danø K, Andreasen PA, Grøndahl-Hansen J, Kristensen P, Nielsen LS and Skriver L (1985) Plasminogen activators, tissue degradation, and cancer. *Adv Cancer Res* **44**: 139–266

De Bruin PAF, Griffioen G, Verspaget HW, Verheijen JH, Dooijewaard G, Van den Ingh HF and Lamers BHW (1988) Plasminogen activator profiles in neoplastic tissues of the human colon. *Cancer Res* **48**: 4520–4524

De Witte JH, Sweep CGJ, Klijn J, Grebenschikov N, Peters H, Look M, Van Tienoven ThH, Van Putten W, Benraad ThJ and Foekens JA (1998) Prognostic impact of urokinase-type plasminogen activator (uPA) and its inhibitor (PAI-1) in cytosols and pellet extracts derived from 892 breast cancer patients. *Br J Cancer* (accepted for publication)

Dudani AK and Ganz PR (1996) Endothelial cell surface actin serves as a binding site for plasminogen, tissue-type plasminogen activator and lipoprotein(a). *Br J Haematol* **95**: 168–178

Duffy MJ (1996) Proteases as prognostic markers in cancer. *Clin Cancer Res* **2**: 613–618

Duffy MJ, O'Grady P, Simon J, Rose M and Lijnen HR (1986) Tissue-type plasminogen activator in breast cancer: relationship with estradiol and progesterone receptors. *J Natl Cancer Inst* **77**: 621–623

Duffy MJ, O'Grady P, Devaney D, O'Siorain L, Fennelly JJ and Lijnen HR (1988) Tissue-type plasminogen activator, a new prognostic marker in breast cancer. *Cancer Res* **48**: 1348–1349

Duggan C, Kennedy S, Kramer MD, Barnes C, Elvin P, McDermott E, O'Higgins N and Duffy MJ (1997) Plasminogen activator inhibitor type 2 in breast cancer. *Br J Cancer* **76**: 622–627

Egelund R, Schousboe SL, Sottrup-Jensen L, Rodenburg KW and Andreasen PA (1997) Type-1 plasminogen-activator inhibitor; conformational differences between latent, active, reactive-centre-cleaved and plasminogen-activator-complexed forms, as probed by proteolytic susceptibility. *Eur J Biochem* **248**: 775–785

EORTC Breast Cancer Cooperative Group (1980) Revision of the standards for the assessment of hormone receptors in human breast cancer; report of the second EORTC workshop, held on 16–17 March 1979, in The Netherlands Cancer Institute. *Eur J Cancer* **16**: 1513–1515

Foekens JA, Portengen H, Van Putten WLJ, Peters HA, Krijnen HLJM, Alexieva-Figusch J and Klijn JGM (1989) Prognostic value of estrogen and progesterone receptors measured by enzyme immunoassays in human breast tumor cytosols. *Cancer Res* **49**: 5823–5828

Grebenschikov N, Geurts-Moespot A, De Witte H, Heuvel J, Leake R, Sweep F and Benraad T (1997) A sensitive and robust assay for urokinase and tissue-type plasminogen activators (uPA and tPA) and their inhibitor type I (PAI-1) in breast tumor cytosols. *Int J Biol Markers* **12**: 6–14

Grøndahl-Hansen J, Bach F and Munckholm-Larsen P (1990) Tissue-type plasminogen activator in plasma from breast cancer patients determined by enzyme-linked immunosorbent assay. *Br J Cancer* **61**: 412–414

Hansson P-O, Eriksson H, Eriksson E, Jagenburg R, Lukes P and Risberg B (1994) Can laboratory testing improve screening strategies for deep vein thrombosis at an emergency unit? *J Int Med* **235**: 143–151

Hekman CM and Loskutoff DJ (1985) Endothelial cells produce a latent inhibitor of plasminogen activators that can be activated by denaturants. *J Biol Chem* **260**: 11581–11587

Jänicke F, Schmitt M, Hafter R, Hollrieder A, Babic R, Ulm K, Gössner W and Graeff H (1990) Urokinase-type plasminogen activator (u-PA) antigen is a predictor of early relapse in breast cancer. *Fibrinolysis* **4**: 69–78

Jänicke F, Schmitt M and Graeff H (1991) Clinical relevance of the urokinase-type and tissue-type plasminogen activators and of their type-1 inhibitor in breast cancer. *Semin Thromb Hemostas* **17**: 303–312

Kaplan EL and Meier P (1958) Non-parametric estimation from incomplete observation. *J Am Stat Assoc* **53**: 457–481

Layer GT, Burnand KG, Gaffney PJ, Cederholm-Williams SA, Mahmoud M, Houlbrook S and Pattison M (1987) Tissue plasminogen activators in breast cancer. *Thromb Res* **45**: 601–607

Levin EG, Santell L and Osborn KG (1997) The expression of endothelial tissue plasminogen activator in vivo: a function defined by vessel size and anatomic location. *J Cell Sci* **110**: 139–148

Maser RE, Ellis D, Erbey JR and Orchard TJ (1997) Do tissue plasminogen activator-plasminogen activator inhibitor-1 complexes relate to the complications of insulin-dependent diabetes mellitus? Pittsburgh Epidemiology of Diabetes Complications Study. *J Diabetes Complications* **11**: 243–249

Mignatti P and Rifkin DB (1993) Biology and biochemistry of proteinases in tumor invasion. *Physiol Rev* **73**: 161–195

Mira-Y-Lopez R and Ossowski L (1987) Hormonal modulation of plasminogen activator; an approach to predicting human breast tumor responsiveness. *Cancer Res* **47**: 3558–3564

Needham GK, Nicholson S, Angus B, Farndon JR and Harris AL (1988) Relationship of membrane-bound tissue type and urokinase type plasminogen activators in human breast cancers to estrogen and epidermal growth factor receptors. *Cancer Res* **48**: 6603–6607

Niwano H, Takahashi H, Tatewaki W, Wada K, Seki Y and Shibata A (1992) Behaviour of tissue plasminogen activator, plasminogen activator inhibitor 1 and their complex in various disease states. *Blood Coagul Fibrinol* **3**: 389–393

Rella C, Coviello M, Quaranta M and Paradiso A (1993) Tissue-type plasminogen activator as a marker of functional steroid receptors in human breast cancer. *Thromb Res* **69**: 209–220

Rijken DC (1995) Plasminogen activators and plasminogen activator inhibitors: biochemical aspects. *Baillière's Clin Haematol* **8**: 291–312

Schmitt M, Jänicke F and Graeff H (1990) Tumour-associated fibrinolysis: the prognostic relevance of plasminogen activators uPA and tPA in human breast cancer. *Blood Coagul Fibrinol* **1**: 695–702

Schmitt M, Harbeck N, Thomssen C, Wilhelm O, Magdolen V, Reuning U, Ulm K, Höfler H, Jänicke F and Graeff H (1997) Clinical impact of the plasminogen activation system in tumor invasion and metastasis: prognostic relevance and target for therapy. *Thromb Haemost* **78**: 285–296

Yamashita J-I, Ogawa M, Yamashita S, Nakashima Y, Saishoji T, Nomura K, Inada K and Kawano I (1993) Differential biological significance of tissue-type and urokinase-type plasminogen activator in human breast cancer. *Br J Cancer* **68**: 524–529

Yamashita J-I, Ogawa M and Sakai K (1995) Prognostic significance of three novel biologic factors in a clinical trial of adjuvant therapy for node-negative breast cancer. *Surgery* **117**: 601–608

 © Cancer Research Campaign 1999
